# Dissection of the internal carotid artery and stroke after mandibular fractures: a case report and review of the literature

**DOI:** 10.1186/s13256-017-1316-1

**Published:** 2017-06-02

**Authors:** Ingrid Aune Tveita, Martin Ragnar Skjerve Madsen, Erik Waage Nielsen

**Affiliations:** 1 Department of Ear Nose and Throat Surgery, Nordland Hospital, Bodø, Norway; 2 Department of Oral and Maxillofacial Surgery, Nordland Hospital, Bodø, Norway; 3 Department of Anesthesiology and Intensive Care, Nordland Hospital, Bodø, Norway; 4 Institute of Clinical Medicine, University of Tromsø, Tromsø, Norway; 5 Faculty of Professional Studies, Nord University, Bodø, Norway

**Keywords:** Blunt cerebrovascular injury, Mandibular fracture, Carotid artery dissection, Facial trauma, Blunt vascular injury

## Abstract

**Background:**

We present a report of a patient with blunt trauma and mandibular fractures who developed a significant cerebral infarction due to an initially unrecognized injury of her left internal carotid artery. We believe that increased knowledge of this association will facilitate early recognition and hence prevention of a devastating outcome.

**Case presentation:**

A 41-year-old ethnic Norwegian woman presented to our Emergency Room after a bicycle accident that had caused a direct blow to her chin. At admittance, her Glasgow Coma Scale was 15. Initial trauma computed tomography showed triple fractures of her mandible, but no further pathology. She was placed in our Intensive Care Unit awaiting open reduction of her mandibular fractures. During the following 9 hours, she showed recurrent episodes of confusion and a progressive right-sided hemiparesis. Repeated cerebral computed tomography revealed no further pathology compared to the initial scan. She had magnetic resonance angiography 17 hours after admittance, which showed dissection and thrombus formation in her left internal carotid artery, total occlusion of her left medial cerebral artery, and left middle cerebral artery infarction was detected.

**Conclusions:**

Carotid artery dissection is a rare but life-threatening condition that can develop after trauma to the head and neck. There should be a high index of suspicion in patients with a mechanism of injury that places the internal carotid artery at risk because blunt vascular injury may show delayed onset with no initial symptoms of vascular damage. By implementing an algorithm for early detection and treatment of these injuries, serious brain damage may be avoided.

## Background

The case presented illustrates the link between facial fractures and blunt cerebrovascular injury (BCI) that, falsely, has long been considered a curiosity. In order to identify these patients at an early stage this case report emphasizes the need for implementation of appropriate screening protocols in the Emergency Room (ER). This was not the case at our hospital at the time the actual patient was admitted, and hence diagnosis was delayed with consequences for patient outcome.

BCI has long been considered a curiosity, and may explain why clinically recognizable neurological symptoms often occur before diagnosis is made [1]. Early reports suggest mortality rates of 28%, and subsequent multicenter reviews have confirmed these rates, with 48 to 58% of survivors having permanent severe neurological deficits [2].

The incidence of BCI among all patients experiencing blunt trauma in the United States of America (USA) is estimated at approximately 0.1%, rising to 1.6% with initiation of screening [2–4].

Early antithrombotic intervention has the potential to improve neurologic outcome, given that BCI is confirmed and that no contraindications for this treatment exist (for example, bleeding pelvic fracture) [5–7].

## Case presentation

A healthy, non-tobacco smoking, 41-year-old ethnic Norwegian woman presented to our ER 10 minutes after a bicycle accident. On arrival, she was conscious and complained of jaw pain. She explained that she, after rapid deceleration, had fallen over the handlebars and landed on her face. She was wearing a helmet. A trauma assessment was initiated: her Airway, Breathing, Circulation, Disability, Exposure assessment and Glasgow Coma Scale (GCS) were normal. A physical examination revealed blood pressure 130/60 mmHg, respiratory rate 20 breaths per minute, and normal auscultatory findings of her heart and lungs. Her blood tests showed normal complete blood count (CBC), bleeding status, and coagulation status, as well as liver and renal function.

**Fig. 1:**
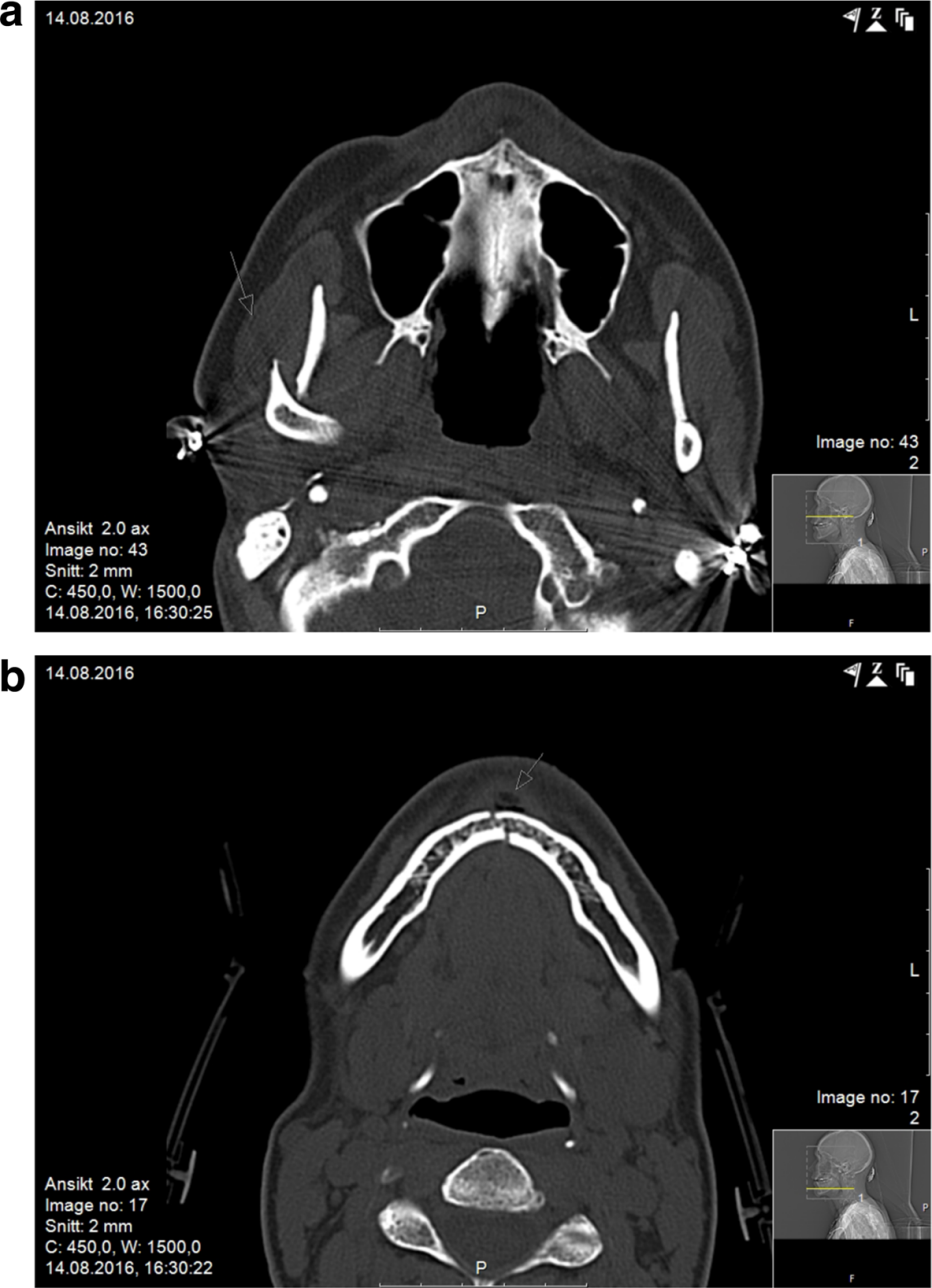
**a**, **b** Computed tomography scan at admittance shows fractures of the mandible (bilateral condylar neck and left corpus paramedian)

A trauma computed tomography (CT) scan reported normal brain status, but fractures of the mandible were found in both condylar necks and the left paramedian corpus (Fig. 1).

She was taken to our Intensive Care Unit (ICU) and, due to stable fractures, the surgery was planned for the next morning. She became confused and had several bouts of tachycardia 1.5 hours after admittance. She responded to verbal contact but was unable to follow instructions. After a few minutes she seemed more alert and took instructions more actively.

A quick neurological examination was made, with the only concern being a slightly impaired finger-nose test on the right side.

The evening passed with a few more episodes of brief confusion.

A repeated neurological examination gave suspicion of brain stem involvement with a decline in GCS to 7 (M 5, V 1, E 1). A repeated CT scan, 10 hours after admission, showed no recent changes. Unfortunately, no further immediate radiologic assessment was initiated.

Her vital parameters were stable; magnetic resonance imaging (MRI) was planned for the next morning.

**Fig. 2:**
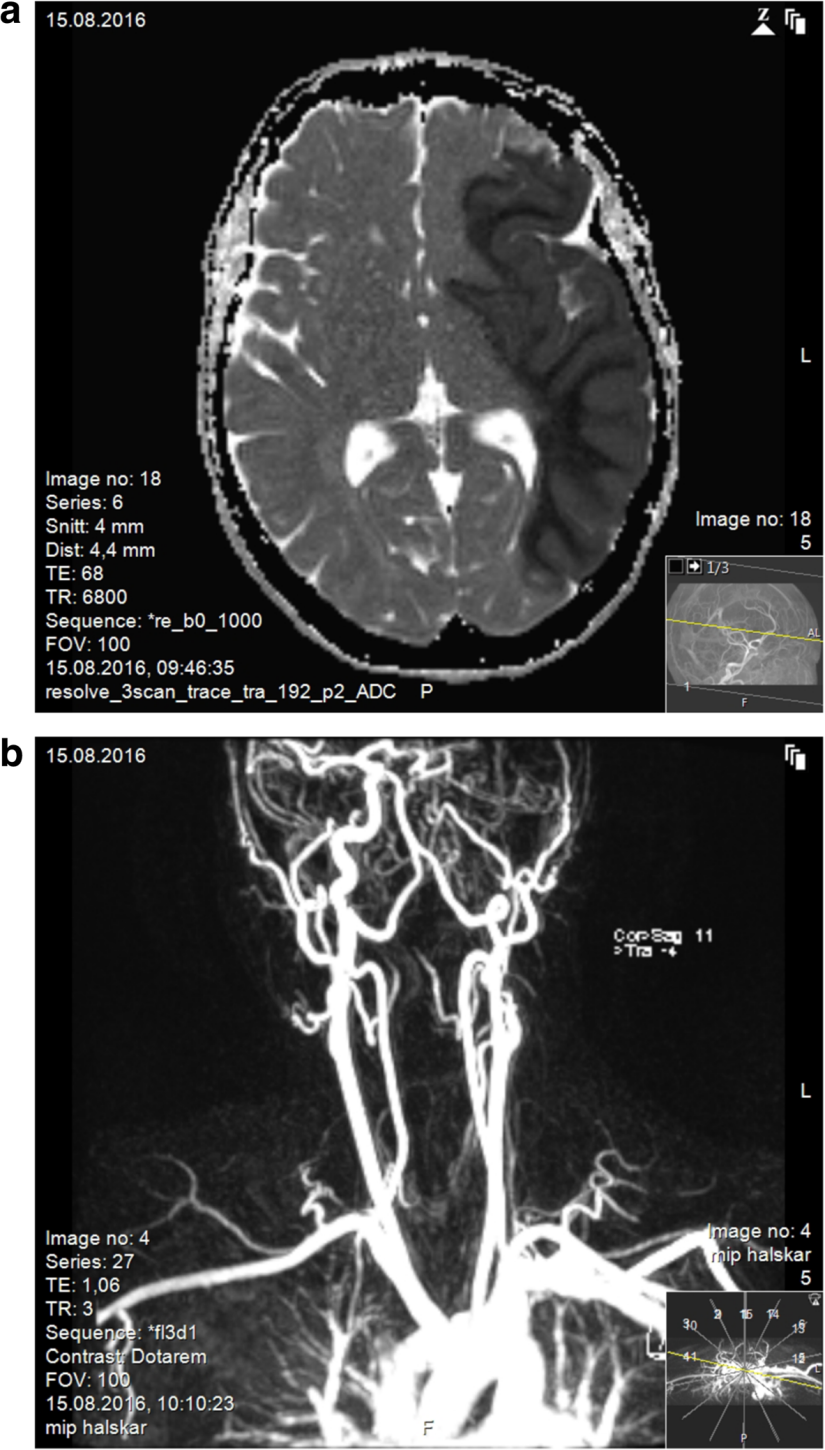
**a** Head magnetic resonance imaging taken 17 hours after admittance (*top*); Shows an extensive left middle cerebral artery infarction. **b** Magnetic resonance angiography taken 17 hours after admittance (*bottom*); Shows an occluded internal carotid artery on the left side approximately 1 cm above the bifurcation, in addition there is occlusion of the left middle cerebral artery

The following morning she presented with palsy in her right extremities and acquired anisocoria with a larger right pupil. She had a GCS of 6 (M 4, V 1, E 1). She was intubated and an MRI and magnetic resonance angiography (MRA) of her head and neck were conducted 17 hours after admission. An extensive left middle cerebral artery infarction was detected, with cessation of diffusion in the supply area of her left middle cerebral artery (MCA). Further, an occluded left internal carotid artery (ICA) was diagnosed, approximately 1 cm above the bifurcation, in addition to occlusion of her left MCA (Fig. 2).

She was given 300 mg acetylsalicylic acid intravenously to reduce risk for progressive thrombosis, and immediately transferred to a level 1 trauma center where she received neurointensive care and a pressure-releasing hemicraniectomy. Postoperatively, CT angiography and ultrasound of her carotid arteries were made and confirmed dissection of her left ICA with total occlusion of the vessel. In addition, dissection and a moderate-grade to high-grade stenosis of 40% of her right ICA was found.

She was treated with dual platelet inhibition, and fixation of her mandibular fractures was postponed. After 4 days, she was extubated and transferred to our neurological department for rehabilitation. The mandibular fractures were fixated 8 days after the initial trauma. She still has aphasia and a right-sided hemiplegia 2 months after the bicycle injury.

The mechanisms of vessel injury in this case are probably a combination of linear and torsional forces due to hyperextension. Direct pressure from her dislocated mandibular bone may have contributed. Bouts of tachycardia could stem from a disturbed carotid body or from cerebral ischemic insults.

## Discussion

In the case presented, a healthy woman presented with clinical signs of rapid deterioration in neurological status 10 hours after admission to our hospital due to a blunt trauma to her chin. In retrospect, the repeat CT scan showing no recent changes was insufficient to rule out cerebrovascular damage, and should have prompted follow-up angiographic imaging. Unfortunately, this was delayed and 17 hours after admission cerebral MRI and MRA detected an extensive left middle cerebral artery infarction caused by a thrombus occluding her left MCA.

Due to the late diagnosis of a BCI, antithrombotic intervention was delayed until 20 hours after admittance.

It is not known whether earlier diagnostic screening and intervention would have altered our patient’s outcome, but the literature covering BCI indicates that early recognition and treatment of this condition have the potential to reduce fatal outcome. Antithrombotic therapy is usually the only treatment option as surgical repair is not possible due to the location of the thrombus. Thus, early antithrombotic treatment of our patient might have halted progression of thrombosis, and thus possibly reduced the chance of development of serious focal neurological deficit.

Searching for case reports on the subject, there are few records of cases with patients presenting clear symptoms of cerebrovascular injury at hospital admittance. Insight of the mechanisms of trauma associated with BCI is thus crucial for all health care providers working with trauma care. This knowledge will raise awareness and ensure recognition of patients at risk for BCI, and prompt early and appropriate diagnostic screening allowing early institution of treatment.

To facilitate our awareness and recognition of this condition, we present a synopsis of a literature search on BCI emphasizing pathophysiology, screening options, and treatment.

### Pathophysiology

High impact trauma to the neck or face is a potential risk for BCI. An increased awareness of this relationship is crucial to facilitate early recognition of the injury and to initiate early intervention to prevent further complications [8].

Four characteristic mechanisms of blunt carotidal injury are listed in Table 1 [9].

**Table 1:** Characteristic mechanisms of blunt carotidal injury [9]

Type	Mechanism
1	Direct application of force to the neck (seatbelt, strangulation, near-hanging)
2	Hyperextension and contralateral rotation of the head and neck
3	Intraoral trauma that affects the internal carotid artery at the angle of the jaw
4	Laceration of the artery resulting from basilar skull fracture

The pathophysiological cascade is believed to be an intimal tear of the artery created by a force that twists or stretches the vessel, or the vessel is impinged against the underlying bone. This forms a thrombogenic surface, platelet aggregation, and the formation of a thrombus that is partial, complete, or with secondary embolization. Over time the intimal tear may cause subintimal dissection of the vessel [2].

Pseudoaneurysms are less common and occur as a result of partial transection of the artery. Free rupture is also reported.

The latency period between the injury and the development of cerebrovascular symptoms is a characteristic feature of BCI, and considered an opportunity for initiation of preventive therapy. Approximately 80% of patients with BCI show no obvious neurologic manifestations at presentation [5]. Studies suggest that 25 to 50% of patients develop symptoms of BCI as late as 12 hours after the trauma [6, 10–13].

### Screening

There seems to be a lack of consensus regarding the optimal diagnostic strategy for detection of BCI, with controversy about the cost-effectiveness of aggressive screening [14]. With the use of well-compiled screening criteria, patients at risk may be identified early, and preventive treatment initiated.

How to recognize patients at risk? During the years 1990 to 1998 Biffl *et al*. performed linear regression analysis of a liberally screened population (*n* = 249), and defined four independent risk factors for BCI. In 1996 they initiated a screening of at-risk asymptomatic patients using arteriography based on these criteria [1].

Using this approach, 85 patients (34%) were diagnosed as having vascular injuries: 65 patients had carotid injuries, 10 had vertebral injuries, and 10 had both carotid and vertebral injuries. Carotid injuries were bilateral in 32 patients. Among 209 asymptomatic patients, cerebrovascular injuries were diagnosed in 57 (27%). This shows a relatively high yield, considering the potentially devastating outcome of these injuries [1].

In 2010 the Eastern Association for the Surgery of Trauma set out to perform a review of all relevant literature concerning management of BCI, and to develop guidelines for screening, diagnosis, and treatment [15].

Screening has clearly increased the number of BCI injuries diagnosed [5–7], and many trauma centers have implemented screening protocols. The optimal screening criteria remain a topic of debate.

In regard of cost-effectiveness, studies have shown screening to be beneficial [14]. The 2016 recommendation of Biffl *et al*. involves an algorithm based on clinical signs and symptoms that prompt immediate diagnostic evaluation and neurovascular imaging [16].

In the absence of prospective, randomized clinical trials, the current recommendations are based on published observational studies only.

CT angiography is believed to be the most reliable noninvasive screening modality. Sensitivity depends on the number of imaging slices, with 16 slices or more needed for consistent correlation with the results of digital subtraction arteriography (DSA) [17].

DSA remains the gold standard of diagnosis, and is indicated when the level of suspicion is high, despite negative initial imaging results. However, arteriography is an invasive procedure and associated with complications [5]. Another aspect is cost and availability. Arteriography should be reserved for cases where it is required for a definite diagnosis or when an appropriately sensitive CT scanner is not available. MRA has poor specificity (67%) and sensitivity (50 to 75%) compared to DSA and is not recommended [6].

### Treatment

Symptoms, site of injury, severity grade of injury, and associated injuries impact the choice of treatment and follow-up strategy. In the absence of contraindication, such as active hemorrhage, an injury grade-specific recommendation for antithrombotic therapy is given [18, 19]. Several retrospective studies have reported convincing improvement in neurologic outcome among symptomatic patients, with a reduction in the occurrence of stroke in asymptomatic patients with BCI receiving antithrombotic therapy compared with those not treated [5, 11, 14, 18].

Heparin is preferred in the acute setting due to its reversibility. There are, however, no randomized trials comparing clinical outcome of different antithrombotic treatment regimens.

For most patients with BCI, inaccessibility of the site of injury precludes direct surgical repair, as the involved vessel is often located at the base of the skull. According to the grading scale for BCI created in 1999 [20], the recommendation is surgical management for patients with accessible Grade II to V BCI; this in agreement with the guidelines of major trauma societies [15, 21].

Follow-up imaging using CT angiography is recommended 7 to 10 days after identification of the cerebrovascular injury, with repeat imaging after 3 months to determine whether long-term antithrombotic therapy is needed.

## Conclusions

BCI, once considered a rare occurrence, has been recognized with increasing frequency in recent years. With the institution of new screening protocols, BCI is now more commonly observed. Early diagnosis and prompt anticoagulation therapy have reduced the occurrence of ischemic neurologic events and disability.

Adequate management requires a high index of suspicion in victims of trauma where the mechanism of injury places the ICA at risk. As illustrated by the case presented here, high-impact trauma resulting in facial fractures represents a risk for BCI. In order to identify these patients at an early stage we emphasize the need for implementation of appropriate screening protocols in the ER [16].

## Abbreviations

BCI: Blunt cerebrovascular injury
CBC: Complete blood count
CT: Computed tomography
DSA: Digital subtraction arteriography
ER: Emergency Room
GCS: Glasgow Coma Scale
ICA: Internal carotid artery
ICU: Intensive Care Unit
MCA: Middle cerebral artery
MRA: Magnetic resonance angiography
MRI: Magnetic resonance imaging
